# Isolation of Madariaga Virus (MADV) in a Horse Coinfected with Equine Infectious Anemia in Venezuela: A Review of MADV Circulation in the Country

**DOI:** 10.3390/vetsci13010071

**Published:** 2026-01-10

**Authors:** Domingo Garzaro, Nardraka Rodríguez, Gladys Medina, Wilmer Alcazar, Marisol Gualdron, José Alejandro Siem, Yoneira Sulbaran, Miguel Barrios, Ferdinando Liprandi, Rossana C. Jaspe, Flor H. Pujol

**Affiliations:** 1 Laboratorio de Virología Molecular, Centro de Microbiología y Biología Celular, Instituto Venezolano de Investigaciones Científicas, Caracas 1020, Venezuela; dgarzaro@gmail.com (D.G.); yfsulbara@gmail.com (Y.S.); miguelnicolasb@gmail.com (M.B.); 2 Instituto Nacional de Salud Animal Integrada, Maracay 2101, Venezuela; nardrakar@hotmail.com (N.R.); wilmeralcazar@hotmail.com (W.A.); gualdronvargasmarisol@gmail.com (M.G.); alesiemagroecologianimal@gmail.com (J.A.S.); 3 Edificio de Sanidad Animal, Laboratorio de Arbovirus-CENIAP/INIA, Maracay 2101, Venezuela; gladicitamedina1@hotmail.com; 4 Laboratorio de Biología de Virus, Centro de Microbiología y Biología Celular, Instituto Venezolano de Investigaciones Científicas, Caracas 1020, Venezuela; fliprand@gmail.com

**Keywords:** eastern equine encephalitis virus complex, Madariaga virus, equine infectious anemia, surveillance, genome sequencing

## Abstract

Background: Madariaga virus (MADV), formerly known as the South American variant of Eastern Equine Encephalitis virus (EEEV), is an alphavirus that belongs to the *Togaviridae* family and has been periodically infecting equids in Venezuela since its first identification in 1975. This study reports the isolation and molecular characterization of MADV isolated from a horse in December 2024 in the context of MADV cases reported in Venezuela. Methods: Antibodies to the rabies virus were detected by indirect immunofluorescence, and to the Equine Infectious Anemia virus (EIAV) by passive immunodiffusion. MADV RNA was detected by qRT-PCR. The sequence of the complete viral genome was obtained by next-generation sequencing. Results: The sequence of this virus was highly similar to that of the only human case of MADV reported in the country in 2016, as well as to a sequence of a virus isolated from a horse in Colombia in 2002. The horse was found to be co-infected with EIAV. Conclusions: The continuous circulation of MADV in Venezuela warrants reinforcing the preventive measures against these alphaviruses, which ignore borders, and may cause important animal and human health concerns.

## Simple Summary

Madariaga virus causes severe encephalitis in equids in South America and may also affect humans. This study reports the isolation and molecular characterization of a Madariaga virus isolated from a horse in Venezuela in December 2024 in the context of the viral cases reported in Venezuela. The sequence of this virus was highly similar to that of the only human case reported in the country in 2016, as well as to a sequence of a virus isolated from a horse in Colombia in 2002. The virus has been found continuously circulating in Venezuela since its first identification in 1975.

## 1. Introduction

Madariaga virus (MADV), formerly known as the South American variant of Eastern Equine Encephalitis virus (EEEV), is an alphavirus that belongs to the *Togaviridae* family. This family includes several viruses of high concern for animal and human health in the Americas, such as Venezuelan Equine Encephalitis virus (VEEV), EEEV, and Western Equine Encephalitis virus [1]. Epizootic outbreaks of MADV have been described in Brazil, Colombia, Haiti, Trinidad and Tobago, and Venezuela, as well as in a significant outbreak in Panama, causing high mortality in equine populations and sometimes neurological disease in humans. Both EEEV and MADV outbreaks are generally less severe than the ones caused by VEEV due to the lack of high viremia in equids and humans [1,2]. MADV infection is considered milder than EEEV infection [3].

MADV has a positive-sense, single-stranded RNA genome of approximately 11.7 Kb, with the first open reading frame (ORF) encoding four non-structural proteins (NSP1 to NSP4, the last protein the RNA-dependent polymerase) and the second ORF encoding five structural proteins (SP: C, E3, E2, 6K, and E1) [4]. MADV and EEEV belong to the EEE complex, with EEEV classified as lineage I in this complex, while three lineages are described for MADV. MADV lineage III is the most widely distributed in the Americas, from Panama to Argentina, and the one circulating in Venezuela [1,3].

The first isolation of MADV in Venezuela was in 1975, in the Southwestern part of the Zulia State, a region neighboring Colombia [5]. Since then, sporadic cases have been detected by active surveillance. The most recent published case was a girl infected in 2016, with a probable exposure in Falcón, a western state of Venezuela [6].

In December 2024, notifications of equids with clinical signs suggestive of encephalitis were made to the Instituto Nacional de Salud Agrícola Integral (INSAI), the Venezuelan institute in charge of animal health surveys. A MADV was isolated from an infected horse, which also presented with evidence of infection with Equine Infectious Anemia. The aim of this study is the molecular characterization of this MADV isolate, in the context of MADV cases reported in Venezuela, since its first identification.

## 2. Materials and Methods

### 2.1. Specimens

At the end of 2024, the INSAI, the institute responsible for animal health surveillance, received notification from the municipalities of Almirante Padilla and Rosario de Perijá in the state of Zulia of presumptive cases of encephalitis and other co-morbidities, particularly in horses. Two visits were coordinated. The first visit was on 22 December and the second on 27 December 2024, where different species were observed, including horses, goats, and sheep, presenting with common signs such as pale mucous membranes, nasal and ocular secretions with a serous–purulent appearance, weakness, difficulty breathing, and abundant ectoparasites. The inhabitants of the area alleged a large plague load (horseflies and mosquitoes). Of the four horses evaluated, one was found in a lateral cubitus with neurological symptoms, and it was kept under observation until its death by the time of the second visit. Blood serum and fecal samples from 12 horses and a goat with symptoms were collected and analyzed for differential diagnosis of rabies and equine encephalitis, in addition to infectious equine anemia and gastrointestinal parasites. Brain tissue of the dead horse was also collected. Following the visit, contact was made with the municipal mayor’s office, and, to date, no further sick or dead animals have been reported. This study was approved by the Animal Bioethical Committee of IVIC.

### 2.2. Detection of Parasites and Antibodies Against Equine Infectious Anemia and Rabies Viruses

Intestinal parasites were evaluated microscopically in the feces of the animals. Antibodies to the rabies virus were detected by indirect immunofluorescence, as described previously [7]. Antibodies to the Equine Infectious Anemia virus (EIAV) were detected by passive immunodiffusion [8].

### 2.3. Detection of Alphaviruses

RNA was extracted with a Qiagen extraction kit from sera and brain tissue (Qiagen, Germantown, MD 20874, USA). A nested RT-PCR generic for alphavirus, targeting a region of the NSP4 gene, was performed as previously described [9]. The presence of VEEV and MADV was also analyzed by qRT-PCR with probes specific for each virus, as previously described [10].

### 2.4. Cell Culture

A brain homogenate was resuspended in RPMI 1640 and clarified for one hour at 14,000 rpm and 4 °C, and filtered through a Millipore filter of 0.22 µm. This supernatant was used to infect Vero cells, as described previously [11]. The cytopathic effect (around 70% of affected cells) was observed within 72 h, and supernatant was collected for future culturing and sequencing.

### 2.5. Sequencing

The RNA extracted from both the brain tissue and the supernatant of the first passage of cell culture was amplified by RT-PCR using the SuperScriptIII One-Step RT-PCR Platinum Taq HiFi System (Invitrogen, Thermo Fisher Scientific, Waltham, MA, USA) and MADV-specific primers, as described previously [6]. A total of 16 overlapping genomic fragments, which cover the entire viral genome, were amplified and subjected to massive next-generation sequencing using the Illumina iSeq 100 platform and the commercial Microbial Amplicon Prep Kit with IDT for Illumina-PCR Indexes Set 1 (Illumina, Inc., San Diego, CA, USA), following the manufacturer’s recommendations. Viral genome assembly was performed using the Genome Detective platform (https://www.genomedetective.com/, accessed on 5 April 2025). The complete genome sequence was obtained for the cell culture material, and an almost complete sequence was achieved for the brain tissue. The sequences were identical in the genomic region sequenced for both isolates. The complete genome sequence is available at GenBank under the accession number PX473114.

### 2.6. Phylogenetic Analysis

The complete genome sequences of MADV and an EEEV sequence used as outgroup were aligned using MAFFT (https://mafft.cbrc.jp/alignment/server/index.html, accessed on 17 August 2025). Evolutionary analyses were conducted using MEGA12 software [12]. The phylogeny was inferred by using the Maximum Likelihood method [13], and the tree with the highest log likelihood (−41,485.35) is shown. The percentage of replicate trees in which the associated taxa clustered together (1.000 replicates) is shown below the branches. The initial tree for the heuristic search was selected by choosing the tree with the superior log-likelihood between a Neighbor-Joining (NJ) tree and a Maximum Parsimony (MP) tree. The NJ tree was generated using a matrix of pairwise distances computed using the General Time Reversible model. The MP tree had the shortest length among 10 MP tree searches, each performed with a randomly generated starting tree. The evolutionary rate differences among sites were modeled using a discrete Gamma distribution across 5 categories (+G, parameter = 13,984), with 52.76% of sites deemed evolutionarily invariant (+I). The analytical procedure encompassed 15 coding nucleotide sequences using 1st, 2nd, 3rd, and non-coding positions. The partial deletion option was applied to eliminate all positions with less than 95% site coverage, resulting in a final data set comprising 10.853 positions.

## 3. Results

From the 12 horses and the caprine evaluated, *Eimeria* sp. and *Trichostrongylus* sp. gastrointestinal parasites were found in all the feces tested (*n* = 13). EIAV antibodies were detected in the sera of the 12 horses, and none of the samples were positive for rabies.

The 12 sera from the horses, including the one from the dead horse, were found to be negative for alphavirus both by the generic alphavirus RT-PCR and by qRT-PCR. In contrast, MADV could be amplified from the brain of the dead horse, both by the generic alphavirus RT-PCR and by qRT-PCR. It cannot be ruled out that other horses might have been infected with MADV, since viremia is not always found during this viral infection [3].

**Figure 1:**
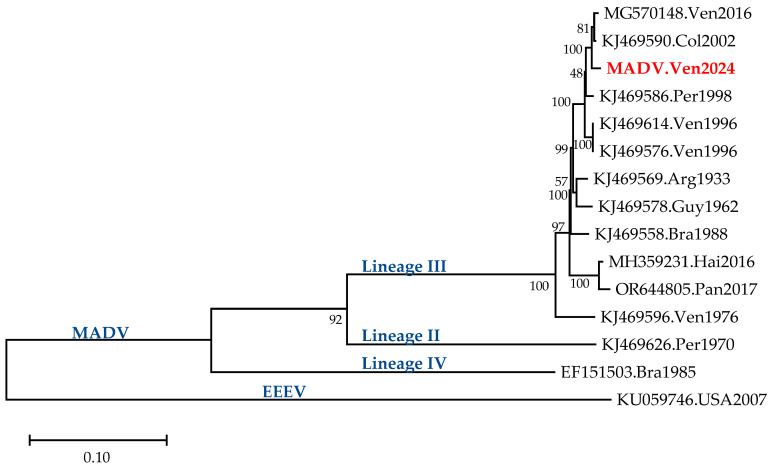
Phylogenetic tree of the MADV Ven 2024 isolate: Maximum Likelihood inference, complete genome. The MADV sequences are named after their accession number in GenBank and the country and date of collection. Sequences of the 3 lineages of MADV and of EEEV (outgroup) are included. The sequence from this study is shown in red.

The sequence analysis of the complete genome of MADV is shown in Figure 1. The isolate was closely related to the one described in the Venezuelan girl infected with MADV in 2016 and to one found in a horse in Colombia in 2002.

Table 1 shows the percent nucleotide identity of the Venezuelan MADV 2024 isolate with respect to the other sequences analyzed. The identity between the sequence from this study and the Colombian 2002 and Venezuelan 2016 ones was higher than 99% (Table 1). A 99.7% protein identity in the NSP region and 99.9% in the SP region was found between this sequence and the MADV.Ven2016 isolate.

**Figure 2:**
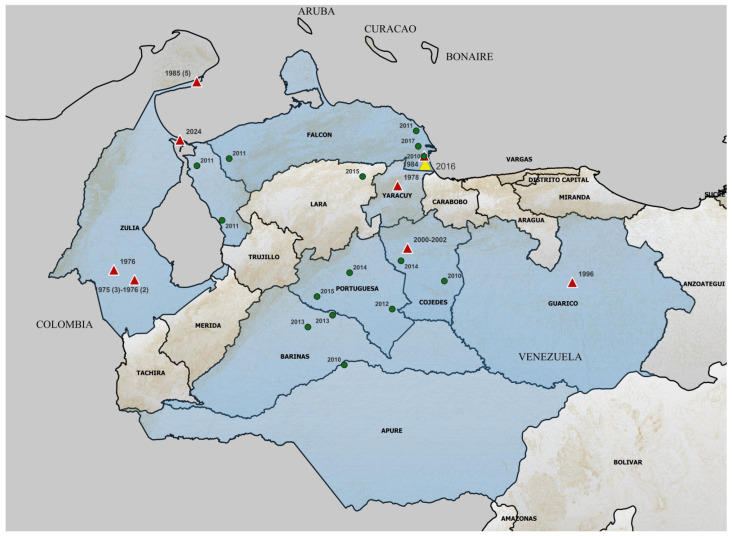
MADV cases in Venezuela since its first identification. The region of Venezuela and where MADV cases have been reported (states with a blue shadow) are shown. Red dots refer to MADV cases with cell culture isolation and green ones to the cases confirmed by serological assays and/or xenodiagnostics [3,5,6]. The yellow dot shows the only known human case in Venezuela. The year of each case is shown for each dot, together with the number of cases under parenthesis if more than one. The positions of the dots were graphed with QGis (https://qgis.org/, accessed on 6 January 2026).

Figure 2 summarizes the reports of MADV confirmed cases in Venezuela, reflecting evidence of active circulation, since the first report in 1975. Almost all the cases have been reported in the north-western region of the country. The Zulia State was one of the states with the highest MADV activity, with two endemic areas, one in the north of the state, and one in the south, both near the border with Colombia. However, both the description of cases and, particularly, the temporal distribution of them may not be a reflection of the intensity of MADV activity in Venezuela, since epidemiological surveillance in the country might not have been constant in each region over time. In any case, the temporal and geographical distribution of MADV cases shows a clear geographical delimitation of MADV circulation in Venezuela.

## 4. Discussion

This study reports the isolation and molecular characterization of a MADV isolated from a horse in Venezuela at the end of 2024, in addition to the historical description of MADV cases reported in the country. MADV was only detected in one of the two brain samples analyzed, and not in any of the 12 serum samples of the horses tested. Considering the clinical signs displayed by all the horses, it cannot be ruled out that many other if not all the horses were also infected with MADV, but the time of collection of serum did not allow us to detect the virus in blood [1,3]. The horses were also found to be infected with EIAV.

Equine Infectious Anemia is caused by a virus (EIAV), *Lentivirus equinfane*, which infects several equine species and is a member of the genus Lentivirus of the family *Retroviridae*. The clinical manifestation can range from mild, almost asymptomatic, to severe thrombocytopenia, anemia, and death, depending on the viral titer and pathogenicity of the strain [14]. Worldwide prevalence of EIAV infection is largely unknown for many countries. A high prevalence has been reported in Mexico [14]. In Venezuela, a single study suggests a relatively high prevalence of 10% [15]. The evidence of infection in all the horses tested in this study was therefore not unexpected. Despite the lack of evidence documenting clinical co-infection between EIAV and equine encephalitis viruses, the epidemiological likelihood of such events is elevated. It is unknown if EIAV infection may predispose horses to a higher susceptibility to MADV infection. As a lentivirus, EIAV infection causes transient immune suppression in horses, with an eventual control of viremia until the virus overcome this control, upon host stress, for example [16]. In addition to EIAV infection, other co-infecting viruses may also have been infecting these horses, such as herpesviruses or papillomaviruses.

The sequence of the Venezuelan isolate displayed high sequence identity with the sequences of a human MADV case from Venezuela and from a horse from Colombia. Although the precise location of the Colombian isolate is unknown, the Colombian frontier with Venezuela has been shown to be an active site of MADV circulation [2]. The genetic relatedness of the three isolates might also suggest a geographical relatedness between them. Transmission dynamics studies of MADV, based on the sequences available in GenBank until 2016, suggested few transmission flows between Colombia and Venezuela [17]. However, the two most recent sequences from Venezuela exhibit a high degree of similarity with the one from Colombia in 2002; transmission over the border of these two countries seems very likely. In addition, we cannot exclude the possibility that other human cases of MADV may have occurred and gone unreported.

The historical description of MADV cases in Venezuela shows a clear regional distribution in the north-western part of the country. Several factors appear to influence the presence of equine cases of MADV in this specific region:

- The presence of vectors: Culex spp. have been proposed, in addition to other mosquitoes such as Aedes taeniorhynchus, as vectors of MADV [1]. Culex spp. have been identified circulating in the north-western region of Venezuela, including states where MADV cases have not been reported, although these are surrounded by states with documented presence of the virus. The case-free states (Tachira, Merida, Trujillo, and Lara, Figure 2) house the Venezuelan mountain “Cordillera de los Andes” [18].
- The presence of reservoirs: Both birds and rodents have been proposed as reservoirs for this virus [1]. A serosurvey in Panama showed that the short-tailed cane mouse (Zygodontomys brevicauda) exhibited the highest levels of seroprevalence against MADV [19]. This rodent is common in the Venezuelan region where MADV cases have been reported [20,21]. Other potential reservoirs include other rodents, marsupials, bats, birds, and even reptiles [19,22]. However, the dynamic and ecological pattern of transmission of MADV is still fairly unknown compared to EEEV. For EEEV, active transmission is limited by the seasonal activity of vectors in the northern part of the USA, while it is continuous in the southern states of the country. EEEV from southern foci is actively transported by infected birds to disperse the virus in the north [23].
- The presence of susceptible equids: The states of Venezuela with confirmed MADV cases have traditionally been associated with equine production [24].

In addition, climate change may expand the geographic range of mosquitoes, including the vectors of MADV, allowing them to invade previously unaffected regions, a phenomenon that is becoming increasingly evident around the world [25].

## 5. Conclusions

As a potential emerging arbovirus pathogen in Venezuela, surveillance programs should be reinforced to include identification of acute MADV cases, as well as serological surveys in equids, particularly in the regions that have historically shown apparent continuous circulation of this virus. More studies are also needed to identify the vectors and reservoirs of this virus in the country. There is no available vaccine against the equine encephalitis alphaviruses for the human general population. Equid vaccination against VEEV is mandatory in Venezuela [26]. Although there is also a vaccine against EEEV for equids, its effectiveness against MADV is still a matter of debate [1]: the only available evidence on the efficacy of the EEEV vaccine against MADV showed that humans immunized with the NA EEEV vaccine did not develop neutralizing antibodies against MADV infection [27]. An effective MADV vaccine for equids is also warranted for the region. The recent epidemic of Western Equine Encephalitis in the South Cone [28,29] is an example of the importance of reinforcing preventive measures against these viruses, which ignore borders and may cause important animal and human health concerns.

## Figures and Tables

**Table 1:** Percent identity of the MADV.Ven2024 isolate with other representative viral isolates.

Virus	Isolate Name ^1^	% Divergence with MADV.Ven2024
MADV Lineage III	MG570148.Ven2016	99.05
	KJ469590.Col2002	99.26
	KJ469586.Per1998	98.52
	KJ469614.Ven1996	98.47
	KJ469576.Ven1996	98.47
	KJ469569.Arg1933	97.40
	KJ469578.Guy1962	97.20
	KJ469558.Bra1988	97.00
	MH359231.Hai2016	96.04
	OR644805.Pan2017	95.60
	KJ469596.Ven1976	95.11
MADV Lineage II	KJ469626.Per1970	83.28
MADV Lineage IV	EF151503.Bra1985	80.50
EEEV	GU001914.USA2003	76.72

^1^ Isolate names as defined in Figure 1.

## Data Availability

The data presented in this study are openly available in GenBank. The complete genome sequence was submitted to the GenBank database with the accession number PX473114.

## Abbreviations

The following abbreviations are used in this manuscript:

EEEV	Eastern Equine Encephalitis Virus
EIAV	Equine Infectious Anemia Virus
INSAI	Instituto Nacional de Salud Agrícola Integral
MADV	Madariaga Virus
ORF	Open Reading Frame
qRT-PCR	Quantitative Reverse Transcriptase Polymerase Chain Reaction
VEEV	Venezuelan Equine Encephalitis Virus
